# Iron Dysregulation in Cardiovascular Diseases

**DOI:** 10.31083/j.rcm2501016

**Published:** 2024-01-10

**Authors:** Hui Wang, Zhongmin Huang, Chenyan Du, Mingqing Dong

**Affiliations:** ^1^Geriatric Diseases Institute of Chengdu, Center for Medicine Research and Translation, Chengdu Fifth People's Hospital, 611137 Chengdu, Sichuan, China

**Keywords:** iron metabolism, homeostasis, cardiovascular disease, dysregulation

## Abstract

Iron metabolism plays a crucial role in various physiological functions of the human body, as it is essential for the growth and development of almost all organisms. Dysregulated iron metabolism—manifested either as iron deficiency or overload—is a significant risk factor for the development of cardiovascular disease (CVD). Moreover, emerging evidence suggests that ferroptosis, a form of iron-dependent programed cell death, may also contribute to CVD development. Understanding the regulatory mechanisms of iron metabolism and ferroptosis in CVD is important for improving disease management. By integrating different perspectives and expertise in the field of CVD-related iron metabolism, this overview provides insights into iron metabolism and CVD, along with approaches for diagnosing, treating, and preventing CVD associated with iron dysregulation.

## 1. Introduction

Iron is an indispensable co-factor for many enzymes and important physiological functions, including oxygen transport and deoxyribonucleic acid (DNA) synthesis [1]. Becouse of it’s essential metabolic role, the body has evolved a complex system to regulate iron’s absorption, utilization, storage, and excretion processes [2]. Accumulating evidences suggest that dysregulation of iron metabolism, including both deficiency and overload, are linked to a range of human diseases, including cardiovascular disease (CVD). Iron deficiency typically arises from inadequate dietary intake, and can result in anemia [3]. Conversely, excessive iron accumulation, typically from iron supplementation or chronic blood transfusions, may lead to organ toxicity and CVD [3, 4, 5]. More specifically, excess iron induces inflammation and mitochondrial dysfunction, factors contributing to arterial sclerosis and heart failure (HF) [5]. Recent studies have highlighted the role of ferroptosis, a newly recognized form of programmed cell death, in the pathology and progression of many CVD, including heart disease, drug-induced HF, and arterial sclerosis. Consequently, effective management and treatment of iron metabolism abnormalities offers a viable approach for the prevention and treatment of CVD.

Iron metabolism is intricately regulated by a network of proteins within the body to balance its absorption and utilization [6]. Sourced mainly from the diet, iron’s complex metabolic pathways have a nuanced relationship with CVD [7]. Hence, understanding this interplay is vital for developing both diagnostic and therapeutic approaches. This review aims to explore current knowledge on the role of iron metabolism in CVD, with particular focus on the mechanisms underlying iron dysregulation and their implications for cardiovascular health. We will also summarize recent advances in treatment strategies for iron-related CVD.

## 2. Iron Metabolism and Homeostasis

**Fig. 1.**
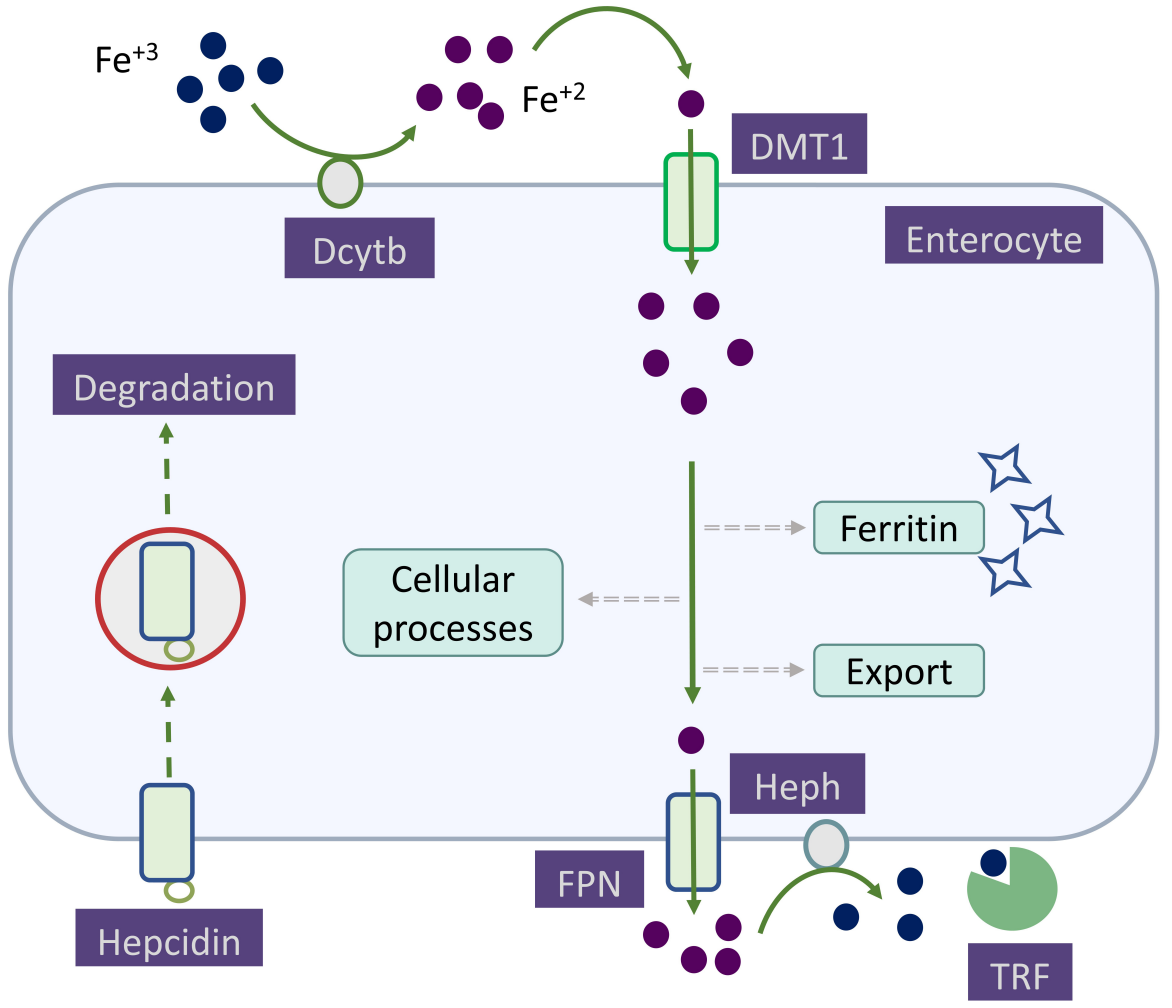
**Non-heme iron absorption by enterocytes**. Duodenal cytochrome B on the apical membrane reduces ${\mathrm{Fe}}^{3+}$ to ${\mathrm{Fe}}^{2+}$ before divalent metal transporter protein 1 transports ${\mathrm{Fe}}^{2+}$ across the apical membrane. Once inside the cell, ${\mathrm{Fe}}^{2+}$ can be stored as ferritin, used in various cellular processes, and transported across the basolateral membrane via ferroportin. In the plasma, ${\mathrm{Fe}}^{2+}$ is reoxidized by hephaestin and, bound to transferrin, delivered to cells expressing transferrin receptors. Hepcidin, by binding to ferroportin, leads to its internalization and degradation, resulting in decreased iron absorption. Dcytb, duodenal cytochrome B; DMT1, divalent metal transporter protein 1; FPN, ferroportin; Heph, hephaestin; TRF, transferrin.

The processes of iron absorption, transportation, utilization, and storage are crucial for maintaining body iron homeostasis. Disorders affecting these processes can disrupt the physiological state of iron [8]. The absorption of iron occurs in the intestines and is affected by both the individual’s iron levels and the type of food consumed. In addition, iron can be aquired from cast iron cooking utensils [9]. Iron has two forms, heme and non-heme, with the latter typically administered orally. Enterocytes in the duodenum and upper jejunum of the small intestine are primarily responsible for the absorption of dietary iron (Fig. 1). By binding to ferroproteins, hepcidin regulates iron absorption based on systemic iron levels [10, 11]. As humans lack an efficient iron excretion system, enteral iron absorption plays a vital role in maintaining an iron balance throughout the body [12, 13].

While absorbed iron is distributed to various body tissues for essential cellular processes, excessive iron selectively accumulates in specific tissues such as the cardiac muscle, adrenal glands, and exocrine glands. In the bloodstream, the majority of iron is tightly bound to transferrin, a protein primarily from the liver. Transferrin facilitates iron transport into cells expressing transferrin receptors, including erythroblasts and macrophages. Therefore, understanding the functional attributes and regulatory mechanisms of transferrin receptors can provide valuable insights into the cardiovascular diseases that may arise from iron dysregulation.

**Fig. 2.**
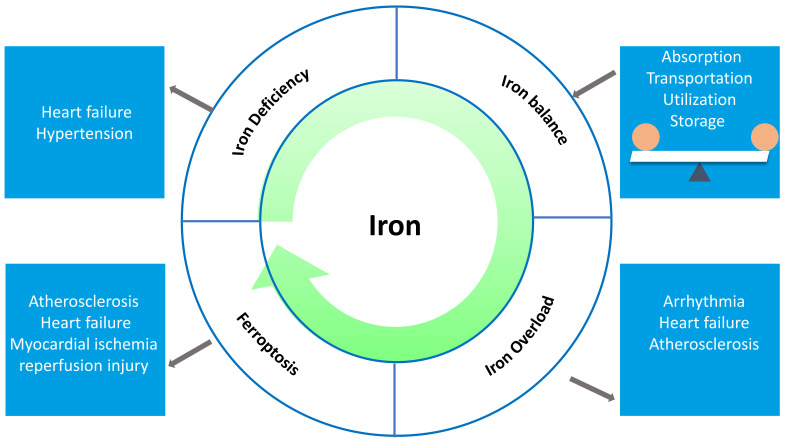
**A potential link between cardiovascular disease (CVD) and abnormal iron homeostasis**. Iron homeostasis is a process of dynamic balance regulated by iron absorption, transportation, utilization, and storage. Disruptions in these process can result in abnormal iron homeostasis, including iron deficiency, iron overload and ferroptosis, and may lead to CVD.

Cellular iron utilization involves processes such as heme synthesis and cellular metabolism, as iron is essential for various catalytic reactions [14]. Intracellular iron exists predominantly as ${\mathrm{Fe}}^{2+}$, while extracellular iron exists predominantly in the form of ${\mathrm{Fe}}^{3+}$. This distinction helps maintain the physiological integrity of cellular compartments, as the cytosol generally has more reducing conditions compared to the extracellular environment [13]. Iron is predominantly found in hemosiderin, with the liver serving as the main place for this storage. Ferritin, mainly presents in tissues like the spleen, meets the body’s demand [15, 16]. Ferritin is the main iron storage protein, which comprises 24 heavy and light chain subunits [17]. *In vitro* studies have shown that the H subunit is necessary for iron uptake, whereas the L subunit facilitates iron core formation inside the protein shell. Fine-tuned regulation of cellular iron ensures an adequate supply of iron to proteins essential for cellular functions. Cells respond to iron restriction through a variety of mechanisms, including upregulating iron import proteins and downregulating iron export and storage machineries [18]. Thus, cellular iron deficiency is less common, and there are few disorders caused by low cellular iron [19]. Surplus iron is peculiarly prone to accumulating in intracellular because humans lack a mechanism for controlled iron excretion. Both cellular and systemic iron overload are associated with several disorders. Additionally, many diseases are characterized by iron overload at the cellular but not at the systemic level [18]. Disruptions in these mechanisms can result in iron deficiency or iron overload diseases, potentially influencing the development of cardiovascular diseases (Fig. 2).

## 3. Iron Deficiency and CVD

**Fig. 3.**
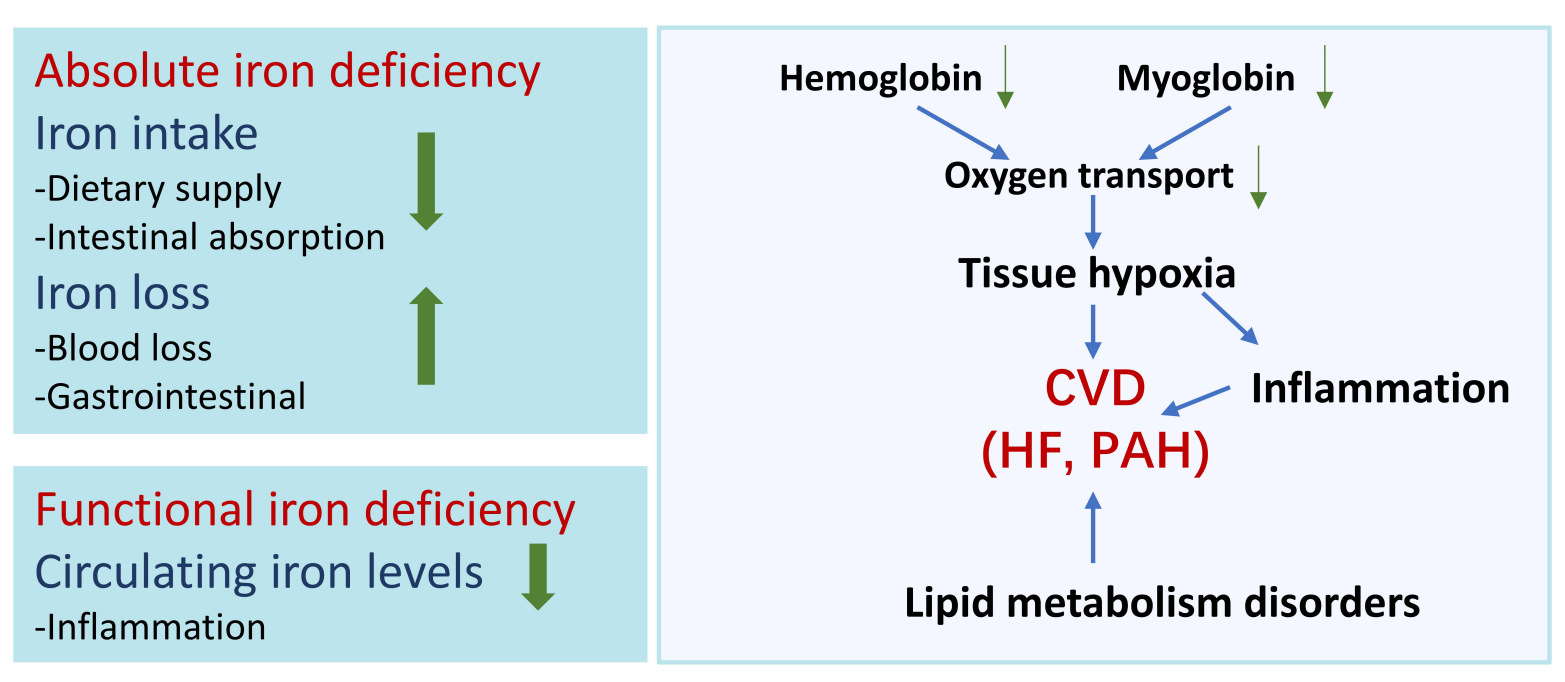
**Iron deficiency and cardiovascular disease (CVD)**. Iron deficiency can be classified into two primary types: absolute iron deficiency and function iron deficiency. Dietary supply and intestinal absorption are the major factor for insufficiency of intake. Cause of iron loss include blood loss and gastrointestinal loss. Inflammation can lead to reduce availability of iron stores even when storage iron is normal. Iron deficiency interfere with hemoglobin and myoglobin function, resulting in tissue hypoxia and inflammation. Additionally, iron deficiency contributes to abnormal lipid metabolism. Iron deficiency is closely associated with the pathogenesis of Heart failure (HF) and pulmonary arterial hypertension (PAH).

Epidemiological studies have demonstrated a strong connection between iron deficiency and CVD. Iron deficiency is common, and recent trials have emphasized its importance as a therapeutic target in patients with CVD, such as HF and pulmonary arterial hypertension [20]. Iron deficiency is frequently observed in various CVD and can be classified into two primary types [21]. The first type is absolute iron deficiency, which is characterized by depleted iron stores owing to insufficient dietary intake or chronic blood loss. The second type is functional iron deficiency, characterized by decreased circulating iron levels associated with chronic inflammatory states commonly observed in different cardiovascular conditions (Fig. 3) [2].

Iron deficiency is the most common micronutrient deficiency in the world, and it is the main cause of anemia [22, 23]. Iron deficiency anemia (IDA) is a global disease, affecting about 1 billion humans, and iron deficiency is likely more prevalent [24, 25]. The non-anaemic iron deficiency (NAID) and IDA are clinically indistinguishable as they share similar symptoms. Strict hemoglobin cut-offs are used to distinguish NAID from IDA at present. Iron deficiency increases the risk of ischemic heart events and cardiovascular mortality. For example, in patients with HF, approximately half of them have iron deficiency, with prevalence estimates ranging from 47% to 68% depending on the definition used [26, 27, 28]. In heart failure, NAID is associated with reduced physical performance, reduced maximum exercise capacity [26] and increased risk of hospital readmission [29]. The correlation between clinical impairments and iron deficiency observed in non-anaemic iron deficient HF patients indicates an important role of iron deficiency. Remarkably, iron deficiency has a stronger causal relationship with hospitalization and mortality than anaemia in HF patients. Hence, iron deficiency seems to have greater clinical repercussions on HF trajectory than anaemia [30, 31].

In patients with advanced HF, there is an increased risk of iron deficiency and anemia, creating a feedback loop that worsens both conditions [32]. Additionally, by mimicking the hypoxia state, iron deficiency can induce inflammation, vasoconstriction and pulmonary vasculature remodeling, resulting in pulmonary arterial hypertension (PAH) [33]. Disabling the transferrin receptor, which facilitates receptor-mediated endocytosis of iron and promotes iron uptake, leads to severe cardiomyopathy, impaired oxidative phosphorylation, and ineffective mitophagy. However, these adverse effects can be rescued by aggressive iron therapy [34]. Several randomized controlled trials showed that intravenous ferric carboxymaltose (FCM) treatment in patients with HF and iron deficiency lead to improved symptoms, quality of life, and functional capacity, irrespective of the presence of anemia [35, 36]. These results suggest that iron deficiency is both a contributing factor and a potential therapeutic target for HF.

Iron deficiency-induced anemia can negatively affect the heart and blood vessels, increasing the risk of CVD. While anemia is more common in children and women, socioeconomic factors and health conditions can make men susceptible as well [37]. Gastrointestinal bleeding can be the usual cause of anemia in women, while reduced dietary iron intake and absorption are contributing factors [38]. As preventing and treating anemia is crucial in managing CVD, addressing anemia has become a significant international health objective. Compromised oxygen transport and altered energy metabolism are thought to be involved in the pathogenesis of CVD caused by iron deficiency. Iron deficiency can lead to lower levels of hemoglobin and myoglobin, impairing oxygen transport and causing tissue hypoxia and dysfunction [39]. Hemoglobin, which relies on iron, plays a key role in oxygen carriage. Decreased iron stores in the body affects hemoglobin synthesis, limiting oxygen delivery to organs. Anemia reduces the blood’s ability to carry oxygen, resulting in tissue hypoxia. Diagnosis is typically based on hematocrit and hemoglobin levels [40, 41]. Iron deficiency is also prevalent in various chronic inflammatory conditions, as well as congestive HF. Moreover, iron deficiency can potentially contribute to lipid metabolism disorders by affecting both lipid synthesis and breakdown, ultimately leading to an elevated risk of CVD (Fig. 3) [42].

## 4. Iron Overload and CVD

While Human cells have multiple redundant iron import mechanisms, there is only one iron-exporting protein, a situation leading increased susceptibility of the heart to iron overload [43]. The pathophysiology of iron overload concerning CVD has attracted significant attention. Excessive iron accumulation in cardiac tissue can cause damage to the myocardium, impaired contractile function, and arrhythmia. Iron overload is characterized by an excessive accumulation of iron in the body, which can have various triggers [44]. Both genetic and acquired factors contribute to iron overload in CVD [45]. Conditions like hereditary hemochromatosis, a genetic disorder characterized by impaired hepcidin activity and increased iron absorption, causing excessive accumulation of iron in tissue [46, 47], can lead to chronic iron overload. This circumstance increases the risk of complications [48, 49, 50, 51]. Other inherited conditions, such as hereditary hemolytic anemias (e.g., thalassemia and sickle cell disease) and acquired hemolytic anemias (e.g., autoimmune hemolytic anemia and myelodysplastic syndrome), can also cause iron overload due to frequent blood transfusions [52, 53]. Transfusion-induced iron overload is especially dangerous in patients with comorbidities. Acquired causes include chronic liver disease, alcohol abuse, excessive iron intake from dietary supplements [54], and end-stage disease [55, 56]. However, the main concern for cardiovascular patients is the potential impact on heart health. The buildup of iron in different organs can lead to more severe symptoms, including HF.

Excessive iron accumulation is a known factor leading to organ dysfunction, with the heart being particularly vulnerable to iron-induced damage [57]. Iron deposits in the heart muscle may lead to an arrhythmia, or heart failure. Iron overload-induced cardiomyopathy is a leading cause of death in both thalassemia and hemochromatosis patients [7, 58]. Moradi *et al*. [59] showed that iron overload, especially in myocardial tissue, is a potential risk factor for ischemic heart disease and acute myocardial infarction . Iron overload has been found to worsen existing cardiovascular conditions [60]. Untreated iron overload can aggravate pre-existing cardiac conditions including myocardial infarction and arrhythmia. Additionally, iron deposition contributes to the formation of plaques, increasing the risk of atherosclerosis. The ATP-binding cassette transporter 8 (ABCB8) is a mitochondrial inner membrane protein involved in mitochondrial iron export. Genetic deletion of ABCB8 in mouse hearts resulted in mitochondrial iron accumulation and cardiomyopathy [61]. Therefore, it is essential to raise awareness of risk factors associated with iron overload when developing effective strategies for preventing and managing CVD. Weakness and joint pain are common symptoms that may indicate iron overload. However, 75% of individuals with iron overload may not experience any symptoms, with fatigue alone serving as an early indicator [62].

**Fig. 4.**
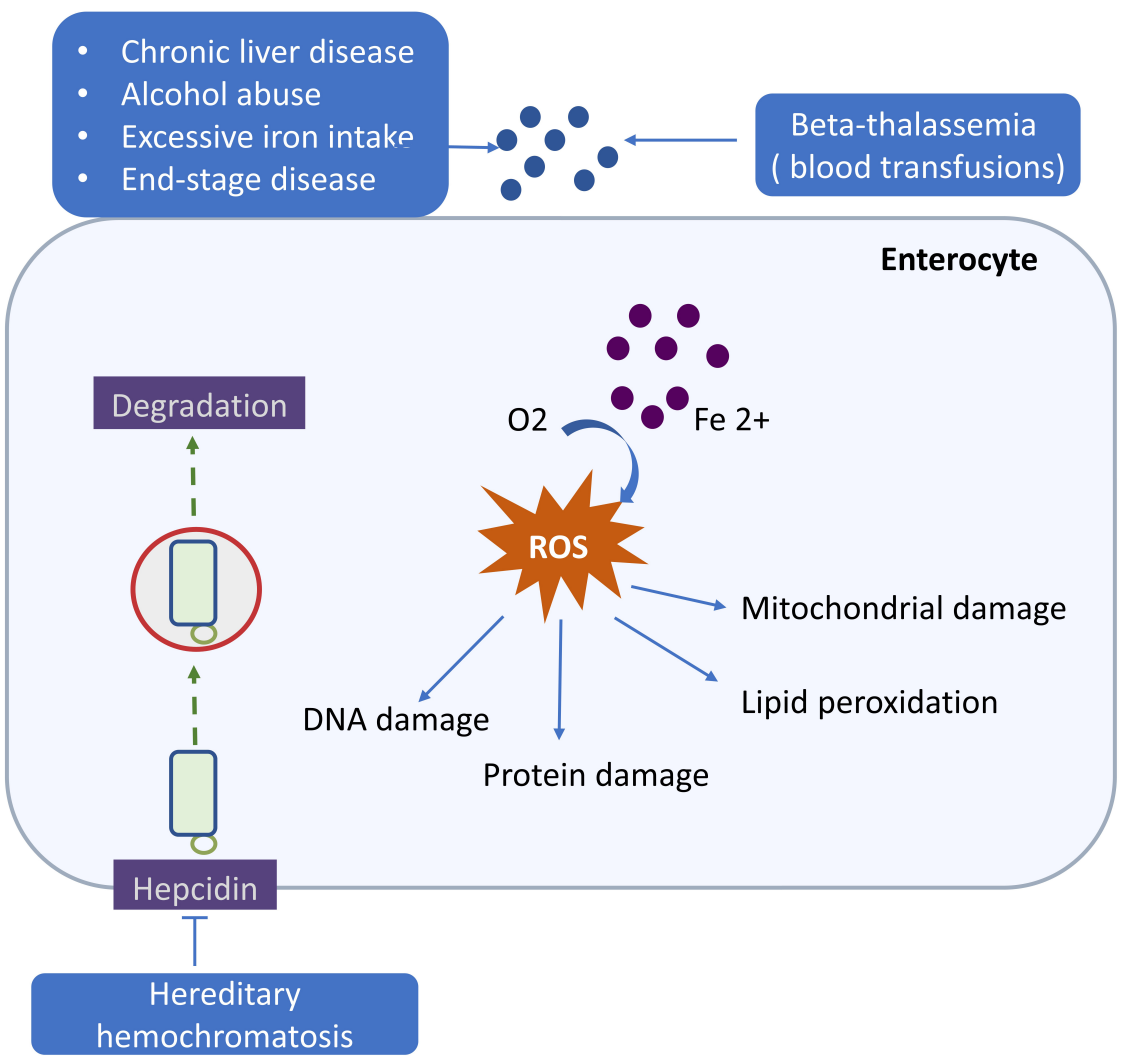
**Iron overload and cardiovascular disease (CVD)**. Both genetic and acquired factors can contribute to iron overload. Hereditary hemochromatosis, a genetic disorder, could cause excessive accumulation of iron by inhibiting hepcidin activity. Iron overload also may occur in Beta-thalassemia who receive regular blood transfusions. The acquired factors include chronic liver disease, alcohol abuse, excessive iron intake from dietary supplements and end-stage disease. reactive oxygen species (ROS) generated by iron overload can damage DNA, protein, lipid peroxidation and mitochondrial. These changes are closely related to CVD.

Oxidative stress, inflammation, endothelial dysfunction, and altered lipid metabolism are potential mechanisms that contribute to the development of CVD. The underlying mechanism is based on iron accumulation causing the activation of multiple signaling pathways and impact cell interactions within the atherosclerotic lesion (Fig. 4). Catalytically active iron is involved in producing reactive oxygen species (ROS) and promoting lipid peroxidation, which is crucial in the development of atherosclerosis [63]. Iron overload is often observed in macrophages and endothelial cells in atherosclerotic lesions. The accumulation of iron can cause endothelial dysfunction by creating pro-oxidant and proinflammatory effects [3]. The excess iron’s cardiotoxicity comes from the production of ROS, leading to oxidative damage [64]. Iron overload not only induces endothelial dysfunction but also promotes the proliferation, apoptosis, and phenotypic switching of vascular smooth muscle cells, contributing to the formation of complex atherosclerotic lesions [65]. ROS generated by iron overload can damage DNA, proteins, and lipid structures in cell membranes, ultimately accelerating cardiomyocyte death. Thus, ROS play an important role in physiological signaling pathways related to cardiovascular tissue injury and disease. Iron overload, complicated by persistent RBC extravasation, results in an increase in proinflammatory M1 macrophages, sustaining inflammation [66]. Uncontrolled macrophage activation is a critical event in the pathogenesis of atherosclerosis. Lipid peroxidation also plays important media role between iron overload and heart injury. When lipid peroxides break down in the heart and plasma, several toxic substances are formed [67].

Iron can impact mitochondrial function, leading to apoptosis and disruptions in cardiomyocyte rhythm. Excessive iron accumulation in the heart can impair its mechanical and electrical functions, leading to heart block and atrial fibrillation in mice [68]. Iron overload has also been linked to systemic hypertension. Arrhythmia is a common complication of iron overload, with prolonged overload resulting in frequent premature ventricular contractions and ventricular tachycardia. These arrhythmias are directly associated with iron accumulation in the myocardium [69]. Excessive production of ROS due to iron overload may trigger the opening of mitochondrial inner membrane anion channels, leading to mitochondrial depolarization and the subsequent release of cytoplasmic anions, which could contribute to arrhythmia [70, 71]. Iron overload mediates cardiomyocyte cell line H9c2 cell death by causing mitochondrial iron accumulation and subsequent general and mitochondrial ROS upregulation. Overexpression of the MitoNEET protein, which deliveres iron between mitochondrial and cytosol, could reduce iron and ROS in the mitochondria [72].

## 5. Ferroptosis and CVD

**Fig. 5.**
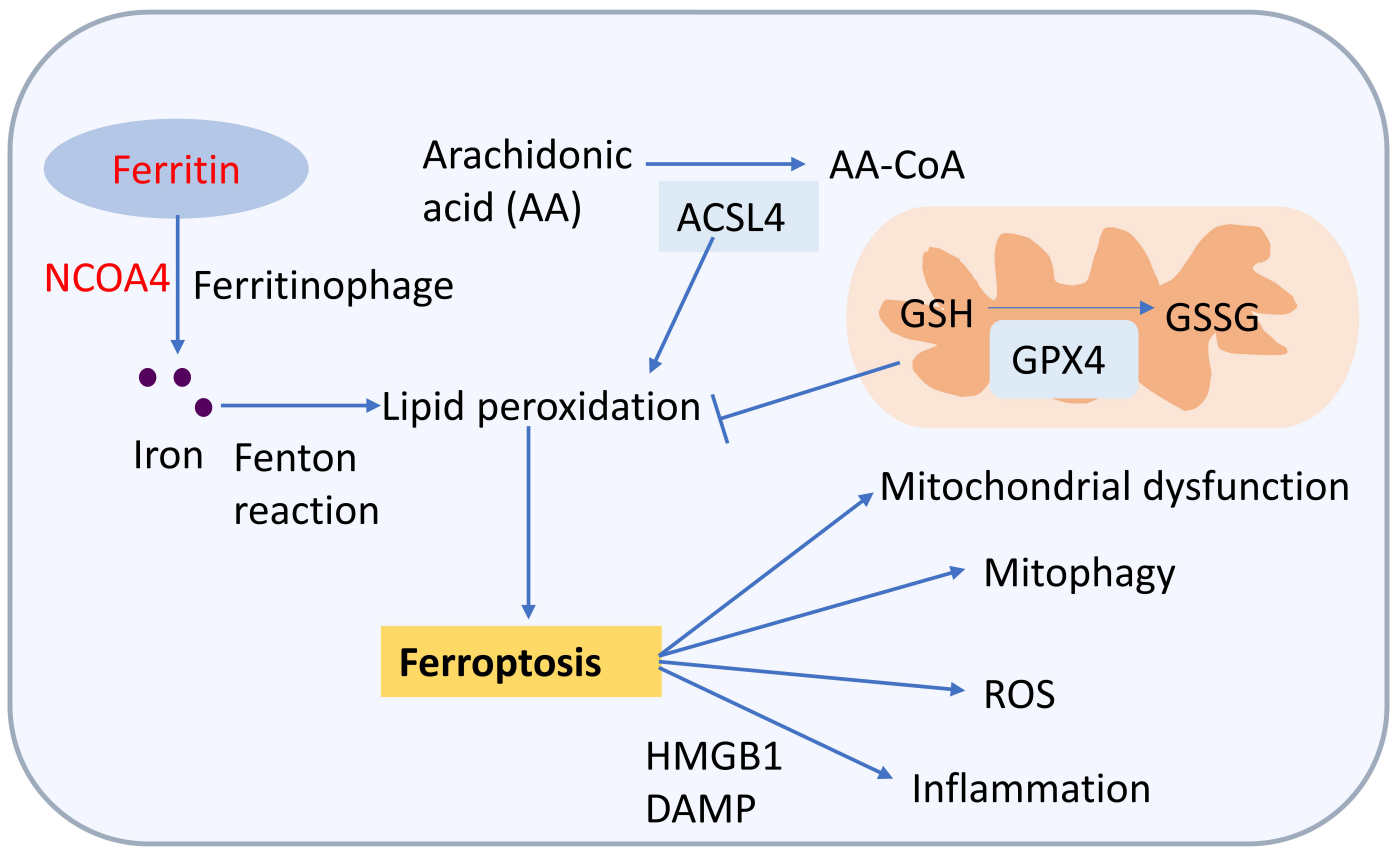
**The ferroptosis and cardiovascular disease (CVD)**. Ferroptosis is an iron-dependent form of programed cell death and driven primarily by iron-dependent lipid peroxidation. Molecules invivoled in iron metabolism and lipid metabolism play an important role in regulating ferroptosis. Iron can be released via nuclear receptor coactivator 4 (NCOA4)-mediated ferritinophagy and promote ferroptosis. The acyl-coa synthetase long-chain family member 4 (ACSL4) promotes lipid peroxidation and ferroptosis. The glutathione peroxidase 4 (GPX4) reduces phospholipid hydroperoxides to their corresponding alcohols by using glutathione (GSH) as a cofactor. Ferroptosis heavily affects many key biological processes and leads to CVD. Ferroptosis can induced inflammation via regulating high mobility group box 1 protein (HMGB1) and damage-associated molecular patterns (DAMP). The mitochondrial function could be affected by ferroptosis. Ferroptosis also cause mitophagy and reactive oxygen species (ROS). GSSG, oxidized glutathione.

Ferroptosis is a newly discovered form of programmed cell death that is iron-dependent and is characterized by iron accumulation and lipid peroxidation [73]. Recent studies have shown that ferroptosis is involved in the pathophysiology of CVD, including atherosclerosis, stroke, HF, and diabetic cardiomyopathy [74, 75]. Iron deposition can trigger ferroptosis, resulting in cardiomyopathy or the formation of vulnerable plaques [76]. Several mechanisms have been proven to be involved in inducing ferroptosis, including mitochondrial abnormalities, glutathione and lipid metabolism (Fig. 5; Table 1, Ref. [68, 69, 70, 77]) [21]. Regulators involved in iron metabolism tightly control ferroptosis due to iron’s involvement in ROS production and enzymatic activity in lipid peroxidation [78]. High levels of ferritin gene expression and large amounts of iron deposition were found in 9.5-day embryos that succumbed to cardiac failure [79]. Glutathione peroxidase 4 (GPX4) can catalyze the reduction of lipid peroxides in cellular membrane. Inhibition of GPX4 function leads to accumulation of lipid hydroperoxides and ferroptosis [77]. Additionally, either genetic knockout or pharmacological inhibition of acyl-coa synthetase long-chain family member 4 (ACSL4), a key enzyme in lipid metabolism, can prevent ferroptosis [80]. Nuclear receptor coactivator 4 (NCOA4) is cargo receptor and delivery of ferritin to lysosomes. Inhibition of the NCOA4 reduces the autophagy degradation of ferritin and ferroptosis [81].

**Table 1.** **Principal modulators involved in ferroptosis regulation**.

Protein	Location	Function	Effects of loss-function or gain-function	Refs.
Glutathione peroxidase 4 (GPX4)	Mitochondrion cytoplasm	Reduces the hydroperoxide group (-OOH) of fatty acids esterified in membrane phospholipids	Inhibition the GPX4 activity leads to accumulation of lipid hydroperoxides and ferroptosis	[68]
Acyl-coa synthetase long-chain family member 4 (ACSL4)	Cell membrane; Mitochondrion outer membrane	Catalyzes the conversion of long-chain fatty acids to their active form acyl-CoA	Knocking out or pharmacological inhibition of ACSL4 prevents ferroptosis	[69]
Nuclear receptor coactivator 4 (NCOA4)	Nucleus	Regulates ferritinophagy	Deletion suppresses erastin-induced ferroptosis and cystine starvation-induced ferroptosis	[70]
Ferritin heavy chain (FHC)	Cytoplasm	Iron storage	Deletion promotes iron-induced cardiac ferroptosis, increases ferroptosis in *Drosophila *and promotes erastin-induced ferroptosis	[77]

Ferroptosis is a likely contributing pathogenetic factor involved in drug-induced cardiomyopathy. Excessive β-adrenergic stimulation induces ferroptosis in cardiomyocytes by changing gene expression of proteings related to iron levels and homeostasis, ultimately leading to cardiotoxicity and structural remodeling of the heart [82]. Ferroptosis has been reported to mainly contribute to DOX-induced cardiotoxicity [83]. Inhibition of ferroptosis by Fer-1 and DXZ treatment prevents DOX-induced cardiac injury. Ferroptosis is strongly linked to DAZ sepsis-induced cardiomyopathy. Studies performed in lipopolysaccharide induced septic cardiomyopathy showed that an increase in NCOA4 expression leads to the degradation of ferritin, iron accumulation, and ferroptosis [84]. Evidence suggests that ferroptosis may also contribute to hypertension by inducing endothelial dysfunction. While the exact mechanism of ferroptosis in CVD is not fully understood, several mechanisms have been proposed, including iron-induced oxidative stress, inflammation, metabolic disorders, and mitochondrial damage (Fig. 5) [76].

The cardiovascular system’s antioxidant framework regulates intracellular ROS and maintains redox homeostasis [85, 86, 87, 88, 89]. Simultaneously, inflammation contributes to CVD through the release of endogenous signaling molecules or damage-associated molecular patterns (DAMPs) from damaged cardiomyocytes [88]. Ferritin heavy chain is an essential mediator of the antioxidant and protective activities of NF-κB via its iron binding activity. Thus, iron metabolism could be a potential approach for anti-inflammatory therapy [90]. Ferroptosis cells release high mobility group box 1 protein (HMGB1) upon certain triggers, activating pro-inflammatory macrophages and microglia. Upregulation of HMGB1 expression is associated with chronic HF and ischemic heart disease [91, 92, 93, 94]. DAMPs can activate inflammasomes, leading to the production of mature cytokines that recruit neutrophils to damaged myocardium, contributing to CVD. Metabolic disorders involving glucose, fatty acid, iron, and mitochondria significantly influence ferroptosis initiation and progression. The glutamine-driven intracellular metabolic pathway plays a fundamental role in cysteine-deprivation-induced ferroptosis [95]. Inhibition of glutamine breakdown reduces heart injury resulting from ischemia-reperfusion [96]. Metabolites from glucose metabolism increase the production of ROS in mitochondria, thereby amplifying the signal for ferroptosis.

Fatty acid metabolism, which serves as the main energy source for the heart, also contributes to ferroptosis in cardiovascular diseases [97, 98]. Metabolites and enzymes involved in fatty acid metabolism can initiate cellular ferroptosis. The pathological process of ferroptosis is not only closely related to ROS [99], but also closely related to mitochondrial dysfunction [100]. Mitochondria are critical for energy production and iron utilization, catabolism, and anabolism. Cellular iron and mitochondrial homeostasis are mutually dependent, as mitochondria rely on iron ions for the synthesis of essential proteins for cellular respiration [101, 102, 103]. Additionally, the mitophagy and ferroptosis have common regulator, such as hypoxia-inducible factor (HIF), NOD-, LRR- and pyrin domain-containing protein 3 (NLRP3) and mammalian target of rapamycin (mTOR) [104]. Noncoding RNAs (ncRNAs), play an important role in the pathogenesis of ferroptosis induced CVD by regulation expression. These ncRNAs regulate the expression of genes associated with different ferroptosis-related events, including iron homeostasis [105], cell protection [106], iron importation, and oxidative-stress attenuation [107].

While reperfusion therapy remains the primary treatment for ischemic cardiomyopathy, its efficacy is limited by myocardial ischemia reperfusion injury (MIRI). Ferritin heavy chain (FHC), the primary iron storage molecule, serves as an essential mediator of the antioxidant and protective activities of NF-κB. FHC-mediated inhibition of JNK signaling depends on suppressing ROS accumulation and is achieved through iron sequestration. In a diabetic rat model, supprressing myocardial ferroptosis has been shown to alleviate MIRI by inhibiting endoplasmic reticulum stress [99]. Additionally, inhibiting glutaminolysis can protect heart tissue from MIRI within an *in vitro* heart model [99]. Research on ferroptosis is still in the initial stage, and several important scientific questions remain to be explored. There are no effective methods to identify ferroptosis, and the link between ferroptosis and CVD remains poorly understood due to the limitation of biomarkers for measuring ferroptosis in patients.

## 6. Management and Treatment of Iron Dysregulation

Early identification and appropriate management of iron dysregulation can help prevent serious complications. Accurate and timely diagnosis involves the analysis of clinical symptoms, medical imaging techniques, and laboratory tests. Magnetic resonance is reliable for this purpose [108, 109]. Laboratory tests can provide quantitative measurements of iron concentration in the bloodstream [110]. The main diagnostic tests include measuring serum ferritin levels and transferrin saturation. In cases where transferrin saturation test results are elevated, a serum ferritin test may be conducted. While increased serum ferritin and transferrin saturation levels typically indicate iron overload, these tests may be influenced by factors such as liver damage or chronic inflammation [111, 112]. Serum ferritin is the most reliable initial test for diagnosing absolute iron deficiency [113, 114].

Treatment options for iron deficiency include iron supplements, dietary interventions, and management of underlying conditions contributing to the deficiency [115]. Consuming foods containing iron can help prevent iron deficiency. Iron supplementation, administered orally or intravenously, depending on the severity, is the main treatment [115]. Patients should be offered dietary advice and oral iron replacement [116]. Intravenous iron should be considered for those patients intolerant of oral iron or with conditions where oral iron is likely to be ineffective. Further investigations are needed if the iron deficiency has not been corrected [117]. Iron supplementation could reduce the subjective measures of fatigue of NAID patients [118]. While it has been suggested that correction of iron deficiency before the development of anaemia may improve symptoms and quality of life [32, 119], there is limited evidence to support this viewpoint. A 6-month randomized, double blind placebo-controlled study showed that combined therapy of a low dose of deferiprone with idebenone improves heart hypertrophy [120]. Careful consideration should be given to iron dosage, potential side effects, and possible drug interactions [121]. Addressing both the underlying condition and the anemia is essential in cases where chronic diseases contribute to iron deficiency anemia, to restore normal cardiovascular function [122]. Large, long-term studies are needed to develop better treatments.

Humans lack endogenous mechanisms to remove excess systemic or myocardial iron. Reducing systemic iron levels or prevent iron entry into tissues is an important treatment strategy for iron overload. Iron chelation therapy serves as the primary treatment for iron overload. Chelating agents like deferoxamine, deferiprone, and deferasirox are used to bind and eliminate excessive iron from the body. Restricting the intake of iron-rich foods can also help manage iron overload. Chelation therapy has shown improvements in patients with iron overload toxicity and cardiac arrhythmia [123]. Therapeutic phlebotomy is an effective method for reducing iron levels, and synthetic hepcidin analogs show promise [124, 125].

Inhibiting cardiac ferroptosis shows promise as a therapeutic strategy in the treatment of cardiovascular disorders. Several targets for inhibiting ferroptosis in CVD have been identified, such as chelators, antioxidants, and Glutathione Peroxidase 4 (GPX4) activators [126]. These targets offer strategies for preventing or reducing ferroptosis-mediated cardiovascular damage. Treatment with recombinant human GPX4 and the ferroptosis inhibitor has been shown to attenuate myocardial injuries [127, 128]. The thiazolidinediones, a class of antidiabetic compound, can ameliorated tissue demise induced by ferroptosis via targeting ACSL4 [80]. Iron accumulation causes endothelial damage, but treatment with the ferroptosis inhibitor has been able to mitigate inflammation and cell death in endothelial cells [106, 129]. As oxidative damage of mitochondria is a major mechanism for ferroptosis-induced heart damage, antioxidant administration could be a treatment choice for ferroptosis induced cardiomyopathy. MitoTEMPO, a superoxide scavenger designed to target the mitochondria, was shown to suppress ferroptosis in cardiac cells and reduced heart dysfunction [130]. Glutaminolysis, the essential component of ferroptosis, may be emphasized as a new therapy target for CVD. Inhibition of glutaminolysis by Compound 968 prevents heart injury induced by ischemia-reperfusion [96]. These findings suggest the potential benefits of targeting ferroptosis in managing CVD through endothelial function modulation. Yet it is worth noting that these findings are based on animal experiments. Further studies examining the clinical applicability of potential targets are needed. Managing and treating iron metabolism is crucial in preventing and treating CVD. Iron chelation therapy, iron supplementation, and dietary modification are valid options depending on the type and severity of iron metabolism disorders. Addressing the underlying causes of iron dysregulation is also critical in CVD treatment (Table 2, Ref. [69, 85, 104, 105, 108, 112, 113, 115]).

**Table 2.** **Management and treatment of iron dysregulation**.

Dysfunction	Targets	Mechanism	Treatment/Drug	Study type	Refs.
Iron deficiency	Level of serum iron	Iron supplements	Supplementation with oral or intravenous iron	Systematic review of randomized controlled trials	[104]
Iron overload	Level of serum iron	Bind and eliminate excessive iron from the body	Deferoxamine deferiprone deferasirox	Randomized double blind placebo-controlled study	[105, 108]
Ferroptosis	Glutathione	Inhibits glutaminolysis	Compound 968	Cell; Mice	[85]
	Antioxidants	Mitochondria-targeted antioxidant	MitoTEMPO	Mice	[115]
	Glutathione peroxidase 4 (GPX4)	Activates GPX4	Dexmedetomidine	Mice	[112, 113]
	Acyl-coa synthetase long-chain family member 4 (ACSL4)	Inhibition of ACSL4	Thiazolidinediones	Mice	[69]

## 7. Current Research and Future Directions

Recent studies on the pathophysiology of iron dysregulation have advanced our understanding of the mechanisms that contribute to the development of CVD. These novel relationships have elucidated the nuanced interactions between iron metabolism and CVD [131, 132]. One current focus of investigation is the role of iron in oxidative stress and inflammation, both of which are key drivers of CVD progression [133, 134]. Studies have demonstrated that excessive iron accumulation can lead to the generation of ROS, resulting in cellular damage and dysfunction [135]. This has paved the way for the development of novel therapies targeting iron-related pathways to attenuate oxidative stress and inflammation in CVD. Moreover, recent investigations have highlighted the impact of iron dysregulation on endothelial function and atherosclerosis. Iron overload has been shown to impair endothelial cell function and promote endothelial dysfunction, a critical step in the initiation and progression of atherosclerosis [136, 137]. Further exploration into the underlying mechanisms of iron-mediated endothelial dysfunction is necessary to identify potential therapeutic targets for preventing or treating CVD.

In addition to understanding the role of iron dysregulation in CVD pathogenesis, future studies should aim to translate these findings into clinical practice. This involves developing iron-targeted therapies or repurposing existing iron chelators to regulate iron levels and alleviate the burden of CVD [138, 139]. Furthermore, studies investigating the potential use of iron markers as diagnostic and prognostic tools in the clinical setting would greatly enhance patient care and management. These advancements go beyond traditional treatment methods, as certain natural bioactive substances have shown preventive effects on iron dysregulation, making them a promising approach for treating iron-related diseases [140, 141]. The field of personalized medicine is also gaining interest, particularly with the use of genetic testing to identify individuals at a higher risk of developing iron overload-induced cardiovascular disease. Considering the influence of individual behavior, nutrition, and drug regulation on iron metabolism, personalized methods of regulating iron metabolism therapy offer distinct advantages for high-risk populations.

## 8. Conclusions

The cardiovascular system requires iron to maintain its high energy demands and metabolic activity. The maintenance of iron homeostasis is essential for proper cardiac function. The human cardiovascular system has multiple redundant iron import proteins and regulatory mechanisms to maintain iron homeostasis. However, humans have only one cellular iron export mechanism, which makes the cardiovascular system, particularly the heart, highly susceptible to iron overload.

Iron metabolism is a critical factor in the development of CVD, with both deficiency and excess posing risks. Therefore, it is essential to understand how iron contributes to CVD damage to develop effective prevention and treatment strategies. Targeting iron metabolism as a therapy shows promise for reducing CVD burden and improving patient outcomes. Additional research is needed to establish the ideal dosage and duration of iron supplementation for individuals with iron deficiency and CVD. Moreover, the safety and effectiveness of iron chelation therapy should be thoroughly evaluated. The field of iron metabolism in CVD is rapidly advancing and has great potential for CVD prevention and treatment.

Understanding the intricate correlation between iron and CVD is crucial for enhancing prevention and treatment strategies. Future research should focus on optimizing methods for assessing and monitoring iron status in both CVD patients and those at risk. Exploring iron-related biomarkers holds promice for early detection and tailored treatment of CVD. Maintaining optimal iron levels is vital to the prevention of CVD. More research is needed to better understand how iron dysregulation contributes to CVD and identify possible intervention targets. This review aims to summarize the current knowledge about the link between iron and CVD and the underlying mechanisms. Enhancing our understanding of the role of iron metabolism in CVD and providing insights into forming therapeutic strategies for preventing and treating iron-related CVD.
